# Introduction of a Framework for Dynamic Knowledge Representation of the Control Structure of Transplant Immunology: Employing the Power of Abstraction with a Solid Organ Transplant Agent-Based Model

**DOI:** 10.3389/fimmu.2015.00561

**Published:** 2015-11-06

**Authors:** Gary An

**Affiliations:** ^1^Department of Surgery, University of Chicago, Chicago, IL, USA

**Keywords:** transplant immunology, agent-based modeling, immunosuppressive agents, mathematical modeling, discrete models, immune system modeling, immune system models, agent-based models

## Abstract

Agent-based modeling has been used to characterize the nested control loops and non-linear dynamics associated with inflammatory and immune responses, particularly as a means of visualizing putative mechanistic hypotheses. This process is termed dynamic knowledge representation and serves a critical role in facilitating the ability to test and potentially falsify hypotheses in the current data- and hypothesis-rich biomedical research environment. Importantly, dynamic computational modeling aids in identifying useful abstractions, a fundamental scientific principle that pervades the physical sciences. Recognizing the critical scientific role of abstraction provides an intellectual and methodological counterweight to the tendency in biology to emphasize comprehensive description as the primary manifestation of biological knowledge. Transplant immunology represents yet another example of the challenge of identifying sufficient understanding of the inflammatory/immune response in order to develop and refine clinically effective interventions. Advances in immunosuppressive therapies have greatly improved solid organ transplant (SOT) outcomes, most notably by reducing and treating acute rejection. The end goal of these transplant immune strategies is to facilitate effective control of the balance between regulatory T cells and the effector/cytotoxic T-cell populations in order to generate, and ideally maintain, a tolerant phenotype. Characterizing the dynamics of immune cell populations and the interactive feedback loops that lead to graft rejection or tolerance is extremely challenging, but is necessary if rational modulation to induce transplant tolerance is to be accomplished. Herein is presented the solid organ agent-based model (SOTABM) as an initial example of an agent-based model (ABM) that abstractly reproduces the cellular and molecular components of the immune response to SOT. Despite its abstract nature, the SOTABM is able to qualitatively reproduce acute rejection and the suppression of acute rejection by immunosuppression to generate transplant tolerance. The SOTABM is intended as an initial example of how ABMs can be used to dynamically represent mechanistic knowledge concerning transplant immunology in a scalable and expandable form and can thus potentially serve as useful adjuncts to the investigation and development of control strategies to induce transplant tolerance.

## Introduction: The Role of Dynamic Knowledge Representation to Address the Translational Dilemma

The central dilemma for the biomedical research community today can be described as a paradoxical challenge of dealing with an embarrassment of riches. Technological advances in experimental methodology have led to an unprecedented ability to probe deeply into the workings of biological systems and acquire information at a level of detail not previously imagined. Advances in computational capability, both in terms of storage and processing, have allowed the analysis of data sets at a fundamentally different scale. However, the challenges of interpreting this plethora of data are growing as quickly as the ability to acquire it. This condition is most evident in the ability to turn this increased basic biomedical knowledge into effective therapies to treat the diseases that most impact society today. The United States Food and Drug Administration report: “Innovation or Stagnation: Challenge and Opportunity on the Critical Path to New Medical Products” (1) clearly delineates a steadily increasing expenditure on Research and Development that is concurrent with a progressive decrease in the delivery of medical products to market; while this report is over a decade old, this trajectory has not substantively changed since the release of that report. This is the *Translational Dilemma* that faces biomedical research: the inability to effectively and efficiently translate basic mechanistic knowledge into clinically effective therapeutics, most apparent in attempts to understand and modulate “systems” processes/disorders, such as sepsis, cancer, wound healing, and immunomodulation (including transplantation). The current situation calls for a re-assessment of the scientific process as currently executed in biomedical research as an initial step toward identifying where and how the process can be augmented by technology. We have asserted that the primary bottleneck in the current biomedical research workflow is the ability to evaluate and falsify the vast sets of putative mechanistic hypotheses being generated from the data-rich environment and that the use of computational modeling for dynamic knowledge representation is the means by which this bottleneck, and the Translational Dilemma, can be addressed (2). With the specific goal of facilitating the computational representation of the mechanistic knowledge generated from basic biological research, agent-based modeling is a modeling method that is particularly well suited for this purpose.

## Dynamic Knowledge Representation with Agent-Based Modeling

Agent-based modeling is a discrete event, object-oriented, rule-based, and often spatially explicit method for dynamic computer modeling that represents systems as a series of interacting components (3–7). An agent-based model (ABM) is a computer program that generates populations of discrete computational objects (or *agents*) that correspond to the component-level at which the reference system is being examined. These computational agents are organized into *agent classes* representing groupings of agents of a similar type defined by shared properties and characteristics. Agents are governed by *agent rules*, which are a series of instructions that allow the agent to be treated as an input–output object. ABM rules are often expressed as conditional statements (“if-then” statements), making ABMs an intuitive way for representing mechanisms identified from basic science research. Consider the following simple example. There is an agent class called cell-type-1 used to represent a particular cell type. That cell type is known to have a particular receptor, which is called receptor-A, which can bind to a ligand, ligand-A. The binding of ligand-A to receptor-A activates a signal transduction enzyme that is called ST-enzyme-B. This knowledge would be expressed in an ABM in the following manner:

Rule for agent-class cell-type-1:

If ligand-A present, then bind to receptor-A

*If receptor-A bound to ligand-A, then activate ST-enzyme*-*B*…

The general nature of a “rule” allows other types of mathematical or computational models (i.e., differential equation, stochastic, or network) to be used as rule systems (7–13). Individual agents incorporate the properties and rule structures of their parent agent class but are able to manifest diverging behavioral paths based on the differing local inputs that are possible through the ABM’s spatially heterogeneous simulation environment. For instance, in the example presented earlier, it can be readily seen that the behavior of different individual computational agents of type cell-type-1 might now deviate from each other: those in the presence of ligand-A will behave differently from those not exposed to ligand-A. This is the key property of ABMs that allow them to behave “realistically,” generating population/system level outputs from the heterogeneous behavioral trajectories of individual agent instances that embody lower-level knowledge and mechanisms. Thus, ABMs intrinsically cross scales of biological organization, utilizing behavioral rules (Scale #1) to determine individual agent behavior (Scale #2) and then aggregating individuals into population dynamics of the global system (Scale #3). The ability to generate distributions of population behavior is also enhanced by the common practice of adding stochastic components to the agents’ rules: this stochasticity may reflect either apparent randomness associated with limitations of measurement, or actual stochastic processes present in the reference system (which may amount to the same thing).

Agent-based modeling has been used in multiple domains, particularly in those systems that can be viewed as involving the interactions between populations of components, such as ecology (14, 15), social/political science (16), microeconomics (17), and epidemiology (18). Agent-based modeling has also been increasingly and more extensively applied to biomedical systems, primarily in terms of characterizing multicellular interactions, such as in the study of sepsis (19–22), cancer (8, 23–26), cellular trafficking (27–31), host–microbe interactions (32, 33), gastrointestinal biology (34–36), and wound healing (12, 37, 38).

By virtue of their rule-based nature, ABMs are an intuitive means of dynamically representing the mechanisms and hypotheses present in the biomedical literature, allowing them to serve as dynamic knowledge representations of mechanistic hypotheses (21, 39). The intrinsic multiscale nature of ABMs allows researchers to translate putative causal mechanisms to system level phenotypes, an essential function in dealing with the complexity of biological systems. Additionally, the non-prescribed nature of the rules embedded in an ABM, which facilitates the initial development of abstract models and the progressive addition of more detail as it becomes needed, makes agent-based modeling well suited as a scalable modular framework that can evolve with the state of knowledge about a particular system (7, 8, 13, 22, 40).

Agent-based models are related to and share many features of other spatially discrete modeling methods, most notably cellular automata. However, what distinguishes ABM from cellular automata and other types of discrete methods is the ease of the mapping between the reference system and the construction of the ABM. Importantly, ABMs facilitate *abstraction*. The process of abstraction is an essential step in the scientific process; it is only through abstraction that generalization is possible: the ability to extrapolate how one seemingly unique object/system can be treated as similar to another seemingly unique object/system. The process of generalization is the means by which science gains its explanatory power: now one thing learned about one object can be applied to another distinct yet related object. It is readily apparent that this principle of abstraction is embedded in the structure of ABMs through the relationship between the descriptions of a particular agent class and the behaviors of the individual instances of that class. Recognizing the essential role of abstraction in the scientific process leads to the driving concept of parsimony in the quest for explanation (i.e., hypothesis construction). Explanatory power is thus tied to an iterative process of evaluating and refining hypotheses that grow from a parsimonious root. The historical, philosophical, and logical bases for this understanding of the scientific process are reviewed and described in Ref. (41). This concept of parsimony also applies to the process of developing computational/mathematical models. As with all mathematical modeling methods, the initial construction of an ABM should keep the rules as simple as possible, often at the initial expense of mechanistic detail. What initially may seem to be a limitation is actually of considerable benefit, as ABMs representing incomplete and uncertain mechanisms can provide a mean of testing the plausibility of those mechanisms (14, 15). As such, the goal of simulation experiments is to provide sufficiently plausible model behavior given a particular ABM such that it is possible to state that the ABM has *face validity*. Face validity is the initial standard for validation as described in the modeling and simulation community and reflects the ability of a particular simulation to behave in a plausible and recognizable way (42, 43). Very often, this is reflected in the qualitative nature of the mapping between the simulation output and the real-world data, with an emphasis on having real-world behaviors targeted at multiple scales. This approach has been termed pattern-oriented modeling (POM) (14, 15) and has an established role in the biomedical application of ABMs (3, 6, 21). While initially developed for the use of agent-based modeling in ecology, the principles of POM, defined as “…the multi-criteria design, selection and calibration of models of complex systems” (14), can serve as a useful framework for the development and use of ABMs in the biomedical context. POM contains three primary elements: (1) patterns used to determine model structure, 2) patterns used for model selection, and 3) patterns used for calibration. Each of these elements is treated with an iterative process that involves identification, instantiating, and refinement. As with all computational models, the greater fidelity of mapping between the ABM and its biological counterparts enhances the correlation between simulation results and the real-world behaviors, but it must be recognized that such increased fidelity can only be achieved through an iterative process of refinement arising from a necessarily parsimonious origin (6, 21).

The advantages of agent-based modeling are most evident when trying to integrate multiple populations of subcomponents (such as biological cells) that interact in a highly dynamic fashion. The multiple cell types and interactions present in transplant immunology represent exactly this type of system. Therefore, presented herein is an abstract representation of fundamental knowledge concerning the process of acute solid organ transplant (SOT) rejection incorporated into the solid organ transplant agent-based model (SOTABM). While there have been multiple prior ABMs of the immune response (as opposed to inflammation) (44–46) to our knowledge, there have been no prior published applications of agent-based modeling to SOT. The fundamental conceptual basis of the SOTABM is the view that effective transplantation centers around a “tipping point” between the proinflammatory aspects of the immune response aimed at eradicating non-self-cells (evolutionarily reflected in infection) versus the anti-inflammatory control mechanisms that prevent that immune response to damaging the host. More specifically, this tipping point is primarily governed by the cellular components that bridge the transition from the non-specific innate inflammatory immune response, which is the primary end effector for cellular/tissue/microbe damage, and the adaptive immune capability that focuses on partitioning response between self and non-self. The SOTABM is intended to provide an initial example of how a dynamic knowledge representation framework can be used to instantiate and replicate the general properties of transplant immunology with respect to acute rejection. As such, the SOTABM necessarily represents a simplified version of the real-world system, with its form the result of modeling choices made by the developer (as is the case with virtually any model, computational, or otherwise) governed by the principle of parsimony. Thus, there is no supposition that the SOTABM is a comprehensive representation of the sum total of knowledge concerning the cellular and molecular mechanisms of transplant immunology. Rather, the situation is quite the opposite, with the SOTABM intended to represent a basic and fundamental set of components and actions sufficient to explain core general behaviors associated with transplant immunology. Furthermore, the SOTABM represents one perspective (the modelers) of what these most basic and fundamental components and actions are. Given the goal of implementing canonical processes, initial models like the SOTABM draw heavily from literature reviews that present the best approximation of what is generally accepted within a scientific community. Therefore, the SOTABM is based on a series of literature reviews of transplant immunology, with particular emphasis of the modeler’s interpretation of Ref. (47) as the central reference text to provide the overall structure of the SOTABM. The exercise of developing the SOTABM as presented in this article is intended as an example of how such biomedical knowledge can be instantiated in an ABM, and in so doing demonstrate how such a process could be extended to incorporate greater mechanistic detail and a wider range of transplant pathophysiology.

Throughout the text we will attempt to clarify the distinction between the actual biological objects and the computational objects used to represent them by depicting the names of the computational objects in courier font.

## Methods

### General Principles and Purpose of the Solid Organ Transplant ABM

As stated earlier, the intent of this presentation of the SOTABM is as a demonstration of how knowledge concerning transplant immunology could be initially incorporated into an ABM, with particular emphasis on the utilization of abstraction and qualitative pattern matching to enhance the understanding of biological systems. It is critical to emphasize the importance of looking at “model” in its verb form: “to model” as opposed to “a model.” As such, one should not think of these models as end products, but rather at subjects for discourse in the iterative process that is science. Admittedly, this viewpoint is not the familiar one for biologists/experimentalists when dealing with “computation” in biomedicine. The far more common perspective is one of the computational modeling as an analysis service rooted in statistics and the identification of correlations: i.e., “Here are my results, tell me what this means using your fancy algorithms.” Alternatively, the use of dynamic computational modeling as a form of knowledge representation and integration (which is how mathematical modeling is most powerfully used in the physical sciences) requires much more engagement on the part of the biologist, where the dynamic computational model is now a “conversation piece,” subject to interactions where its explanatory power is assessed, its underpinnings challenged, and refinements applied, with the intent of moving toward a greater understanding of the system being studied. The SOTABM is very much intended to be the initial step in such an engagement. In reviewing the development and evaluation of the SOTABM presented in this article, the reader is encouraged to note the specific inclusions and omissions made (inherent in the modeling process) and consider how they would potentially address their perceived shortcomings/limitations of the SOTABM if they were to undertake such an exercise. At a fundamental level, dynamic models such as the SOTABM should not be viewed as end products, but rather as objects intended to generate discourse and simulate an iterative process of testing, falsification, and refinement.

#### A Note on Parameters

As with all computational models, ABMs require the use of multiple parameters (constants utilized in the model’s rules). For example, in the sample rule previously provided, its implementation would be:

If [some value of] ligand-A is present, then bind to receptor-A [to some degree].

If [some threshold value] of receptor-A is bound with ligand-A, the activate ST-enzyme-B [to some degree]

It should be immediately evident that the behavior of any model is heavily dependent upon the parameters chosen. As such, the issue of parameter selection holds particular importance in the development and use of computational models. Ideally, choosing parameters that are derived from experimentally available data substantially enhances the believability of a computational model (assuming it behaves plausibly with those parameters). However, the process of experimentally acquiring specific parameters is often extremely difficult, if not infeasible or impossible, given current experimental and sampling technologies. This latter condition, in fact, has substantially limited the adoption of dynamic computational models in biomedical research, where a very stringent and restrictive criteria for what constitutes a “believable model,” dependent upon quantitative parameter and model behavior matching, substantially reduces the number of “believable models” that can actually be produced. Interestingly, it has been argued that such specifically detailed parameters can only be obtained in highly constrained and artificial experimental conditions, with the end result of a model “valid” for those experimental conditions, but of limited applicability beyond those conditions when more systems-level phenomena are being examined (6). This latter understanding is actually more in keeping with the traditional scientific goal of discovery and establishing generalizing principles, as opposed to the engineering paradigm of optimization and design that underlies many researchers’ experience with modeling and simulation.

The demonstration of agent-based modeling with the SOTABM takes the generalizing, parsimonious approach. As noted earlier, one of the advantages of agent-based modeling is its embracement of abstraction as a means of dealing with incomplete knowledge. By utilizing population effects as their primary output metrics, ABMs allow the characterization of system behavior in a more qualitative fashion, at least in the initial stages of development. For this reason, POM (14, 15) and the use of face validity as assessment criteria (42, 43) are heavily utilized in the development and evaluation of ABMs. This shifts the utility of dynamic computational modeling from quantitative prediction or engineering optimization to explorations of plausible and recognizable behaviors; this shift in the goal of modeling influences the selection and determination of the parameters used in the SOTABM. As can be seen in the sample rule explained earlier, ABM rules can start off as logical statements; the addition of conditional reified modifiers turns these rules into expressions closer to arithmetic. This allows certain types of parameters, specifically those associated with processes with known time scales, to be derived arithmetically. Even though these rates are potentially extractable and knowable, within the context of the specific ABM, their actual values are not important. In fact, since the SOTABM utilizes an abstract representation of space, an attempt to directly apply experimentally derived numerical values for those parameters could potentially foster the belief that the model is somehow more “real” than it actually is. Rather, the relationship certain parameters have to other connected parameters is what is crucial for determining the behavior of the model. The relative dependencies of these connected and related parameters can prove very challenging if one required quantitative fidelity, but given that the current modeling goal is determining and examining sets of overall system behaviors, this type of parameter representation is appropriate [and arguably more relevant to translating the findings of a model beyond its specific implementation (6)]. As such, the establishment of these parameter values often starts with an arbitrary range and is generally followed by a heuristic, hand-fitting process involving repeated runs of the ABM and adjustment based on plausible behavior. If such plausible behavior cannot be generated, then this points to a fundamental insufficiency in the model. This process is integral to the development and calibration of an ABM. However, note that this hand-fitting process of calibration occurs before the execution of the presented simulation experiments; there is no retrofitting of parameters based on the outcomes of the actual experimental simulations.

### SOTABM Overview

SOTABM is an abstract representation of the inflammatory and immune components involved in the acute rejection process of a SOT. The SOTABM is implemented in the freeware agent-based modeling toolkit Netlogo (48). Netlogo is a self-contained toolkit for agent-based modeling and is specifically designed to allow non-computer programmers/mathematicians to create dynamic models of their systems of interest. Interested readers are directed to the Netlogo website (https://ccl.northwestern.edu/netlogo/) to see examples and download the toolkit for their own use. Cellular components are depicted by computational agents (“turtles” in Netlogo terminology): some of these cell types are able to move while others remain static. The background grid spaces (“patches” in Netlogo terminology) represent the extracellular environment of the model. Agents hold variables representing determinants of their internal state (i.e., molecular components of the cells), which in turn govern their state transition rules (i.e., behavior). Patches hold variables that represent extracellular mediators, which diffuse between discrete patches using Netlogo’s diffuse function [which takes the value of the variable on an individual patch and evenly distributes some fraction of that value to the surrounding eight patches; see Ref. (48)]. Interactions with the SOTABM take place through the standard Netlogo interface, consisting of various GUI buttons, switches, and sliders by which certain functions are called and parameters set. The stochasticity in the SOTABM is produced by the use of Netlogo’s random number generator to add probabilistic modifiers to the agents’ state transition rules; Netlogo uses the Mersenne Twister pseudorandom generator, one of the most commonly used pseudorandom number generators utilized in software design (48). Consistent with the general modeling strategy that it is necessary to represent the baseline healthy state with some degree of the system robustness and function present in the real-world reference system, the SOTABM is constructed to be able to utilize its inflammatory and immune functions to deal with both sterile injury (i.e., tissue trauma) and an infectious insult. The SOTABM is available for download from http://bionetgen.org/SCAI-wiki/index.php/Main_Page.

### Description of the Model World for the SOTABM

**Figure 1:**
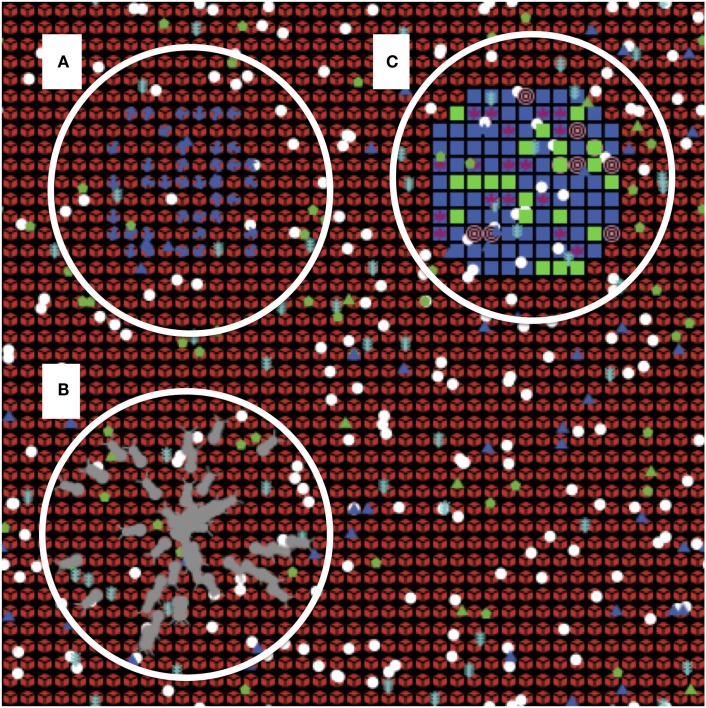
**Screenshot of the solid organ transplant agent-based model (SOTABM)**. This figure depicts the Netlogo graphical interface of the SOTABM. The model world consists of a two-dimensional square grid that is 41 × 41 grid spaces in size. Each grid space is populated by a self-cell (red), and multiple inflammatory/immune cells can be seen distributed over its surface. Letter **(A)** emphasizes the simulated lymph tissue area in the left upper quadrant of the SOTABM; naïve-CD8-ts (blue dots) can be seen in this area. Letter **(B)** emphasizes the region where either infectious insult (here depicted as gray bugs) or tissue trauma (not shown) can be applied. Placement of the perturbation in this area constitutes a “remote” insult from the area of potential transplant. Letter **(C)** emphasizes the area where transplant tissue is applied in the right upper quadrant. Transplant cells can be seen as blue squares, with various other agents representing graft/donor macrophages and dendritic cells can be seen overlying the transplant tissue. Note that the concurrent presence of infection and transplant tissue is provided for depiction purposes only; in the simulation experiments presented for the SOTABM, there were no concurrent types of system perturbations performed.

At its current level of abstraction the SOTABM does not explicitly represent tissue or organ architecture but instead utilizes an abstract representation of various tissue compartments where different cellular interactions occur. The SOTABM does not include the means to differentiate the various degrees of immunogenicity seen between renal, hepatic, and cardiac transplants. The primary interaction space in the host tissue is represented by a two-dimentional square grid where the edges “wrap,” making it topologically a torus. The size of the grid is 41 × 41 grid spaces; this size was arbitrarily chosen to trade off computational efficiency versus enough space to allow for distinct groupings of agents (see Figure 1). Each grid space is populated by an agent representing a generic host tissue cell (self-cell), and populations of immune cells move in a semi-Brownian fashion over this surface. The specific cell types and produced mediators represented in the SOTABM are described later in the respective Section “Methods.” The modeling choice was made to divide the overall world space of the SOTABM into four quadrants each representing a spatially distinct, but still connected, interaction space with different functions. Thus, the SOTABM has a distinct area in the left upper quadrant of the grid, which is intended to represent the intralymph node interaction space in a more spatially defined and limited area. Similarly, simulations of transplanted tissue, as well as remote tissue infection or injury, are localized in different quadrants of the grid (see Figure 1). Three different conditions are able to be applied to the system: Condition #1 sterile injury, Condition #2 localized infection, and Condition #3 solid tissue transplant. Conditions #1 and #2 can be varied in their size and are depicted as generally circular areas; Condition #3 is of fixed size consisting of 109 transplant cells in a roughly rectangular configuration. The size of the simulated transplant (109 cells) is semiarbitrary, decided upon primarily based on the size of the world grid (itself an arbitrary constraint) and the modeling decision to represent different body compartments/tissues in different quadrants of the world grid. As noted earlier, the current version of the SOTABM uses a generic “transplanted tissue,” and therefore does not distinguish between the different immunological properties seen between renal, hepatic, or cardiac tissues. In addition to depicting generic transplanted organ tissue, graft mesenchymal stromal/stem cells (graft-MSCs) are also included in the transplanted area. These cells were selected for inclusion based on their role in suppressing the generation of cytotoxic immune cells directed against the graft (see later). Other graft-associated immune cells, such as macrophages, dendritic cells, and T-cell subtypes residing in any graft lymphoid tissue, were not included since the intent at this stage is not to attempt to represent graft versus host disease. While it is potentially possible to have concurrent conditions within the SOTABM, such as simulating the tissue trauma of transplant followed by the transplanted organ itself, or the development of an infection in a previously transplanted case, for purposes of this initial demonstration of the SOTABM it was elected not to add this complexity at this time.

Given the abstraction of the functions represented in the SOTABM, it is not precisely calibrated to time at a granular mechanistic level. Rather, the effects of the cellular-molecular events are simulated to take place with one cycle of the SOTABM (ticks in Netlogo terminology) approximating 15 min of real-world time.

### Interaction and Control Structure of the SOTABM

**Figure 2:**
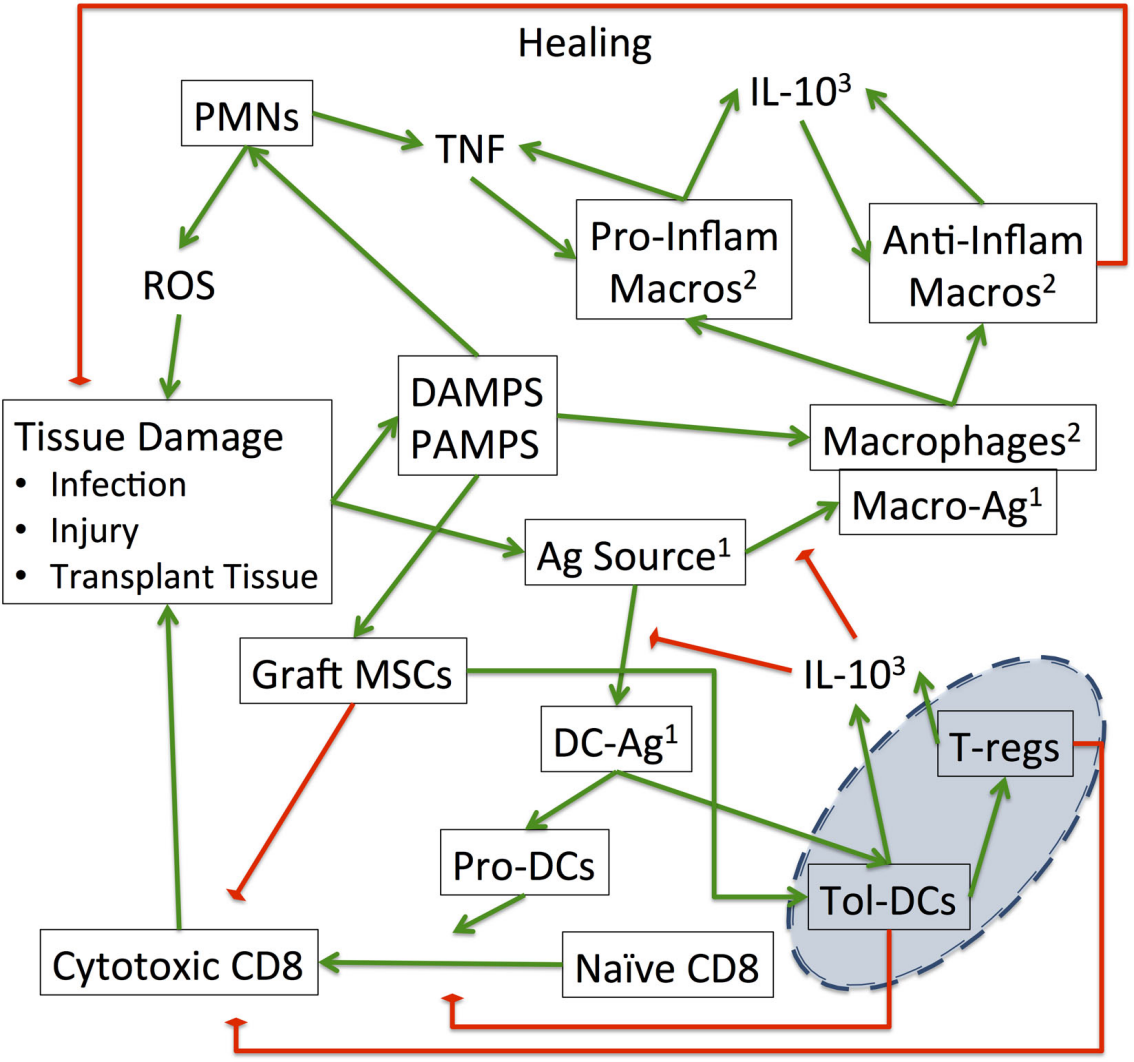
**Interaction map of the SOTABM: this schematic demonstrates the interactions between the various cell types and tissue conditions present in the SOTABM**. The green arrows represent positive/additive/stimulatory relationships, whereas the red-diamonds represent negative/reducing/inhibitory relationships. Note the blue emphasis area in the right lower corner of the figure: these are the primary effector components for tolerance. Augmentation of the functions of T-regs and tolerogenic dendritic cells is a primary goal of immunosuppressive therapies aimed at “tipping” the balance of the control structure depicted here toward the tolerance phenotype (47, 58–60). Notes: ^1^“Ag Source” can come from either infection, injury, or transplanted tissue and has two distinct functional roles, one as an activator for macrophages “Macro-Ag” and one for dendritic cells “DC-Ag.” In the SOTABM, bacteria-Ag represents “Ag source” from infection, whereas transplant-Ag represents “Ag source” from the transplant graft. Both of these bacteria-Ag and transplant-Ag may activate macrophages (as “Macro-Ag”) and dendritic cells (as “DC-Ag”). ^2^The macrophages represented in this figure “Macrophages,” “Pro-Inflam Macros,” and “Anti-Inflam Macros” may be in either the host or the grafted tissue, but in the current iteration of the SOTABM only host macrophages are represented. ^3^The fact that “IL-10” is listed twice is not meant to represent two distinct pools of IL-10 but is rather an attempt to limit the number of crossing connectors in an already complex figure.

As noted earlier, the primary goal of the SOTABM is to serve as an initial example of how to depict the general control structure of the transplant immune response, particularly pertaining to acute rejection, in an ABM. A schematic of this control structure can be seen in Figure 2. Note that in order to not generate a completely uninterpretable figure, the exact model components utilized in the SOTABM (i.e., the names of all the agent classes) are not explicitly represented in Figure 2; rather representative labels are used to depict the main categories of cells and mediators chosen to be included in the SOTABM. Text contextualizing the specific model components to Figure 2 is provided in the descriptions of those components in the sections later. As a general description, the initial components of the innate immune response represent the end effector of the system, being primarily responsible for interactions influencing tissue damage, microbial killing, and abstracted tissue reconstitution. The innate immune response incorporates both pro- and anti-inflammatory components, consistent with a self-contained control structure befitting its role as a highly evolutionarily conserved, fundamental function of multicellular organisms. This component of the SOTABM is very similar to structure to our prior work modeling the acute inflammatory response (20, 22). The SOTABM also includes an additional layer of control representing the regulatory role of lymphocytes, primarily T-cell subtypes. It is noted again that as with all mathematical/computational models, the specific components included in the SOTABM are the result of choices made by the modeler. Given the intent to start from the most well-established and generally accepted components and mechanisms present, introductory ABMs such as the SOTABM often focus on utilizing the content of well-respected review articles for their initial structure. In this case, the initial version of the SOTABM takes the information reviewed in Ref. (47) as its primary source for its included components and mechanisms. While it is clearly evident both from Ref. (47) and from other resources utilized for the development of the SOTABM (49–57) that there are multiple subtypes of regulatory T cells (T-regs), for simplicity’s sake this initial version of the SOTABM abstracts these into the general classes of effector/cytotoxic T cells and T-regs. This simplifying process utilizes the following general guidelines:

1. If a cell type or subtype has essentially the same set of input and output relationships as another cell type, then these were aggregated to a more general cell type description.
2. If a cell type did not have an output relationship that rendered it unique, it was not included.
3. If a cell type served as an intermediate pathway that was otherwise represented in the model using previously selected cell types, it was not included.
4. If a cell type had an output that did not fit into the existing level of functional representation of the SOTABM, such as ischemia–reperfusion, these cells were not included.
5. In general, specific secreted mediator/cytokine relationships were abstracted out if their action could be represented with a cell-to-cell influence/interaction. Note that this does not mean the interaction represented is an actual cell-to-cell physical event, but rather that the effect of the omitted mediator could be represented through a direct relationship.

For instance, comparing Figure 2 with Figures 1 and 2 from Ref. (47), Figure 2 in this paper aggregates T-cell subtypes that have the same input/output or target into the more abstract grouping. This modeling decision is based on the assessment that the impact of the subtleties associated with the finer control provided by these T-cell subtypes is below the representational resolution of the current SOTABM. It is also assumed that there is essentially no lymphoid tissue in the transplanted graft; this is generally consistent with the most common types of SOTs (hepatic, renal, and cardiac) and is consistent with the previously noted decision not to model graft versus host disease at this stage. It is acknowledged that these abstractions may have an impact on the subsequent iterations of the SOTABM as it becomes more refined, but these are accepted possibilities intrinsic to the iterative nature of model development. Furthermore, recognition of these abstractions/omissions would be natural points for future expansion of the SOTABM.

### Cell and Agent Types

The following sections describe the specific agent classes included in the SOTABM. As noted earlier, the overall relationships between the cellular components included are depicted in Figure 2, albeit with a generalization of the cellular subtypes necessary to facilitate depiction in the figure. The relationships and interaction rules for the agents are described later, with the recognition that the encoding of those rules in the SOTABM follows the process described in Section “*A Note on Parameters.” Since the actual values used in the SOTABM would have little meaning outside the context of the actual code, the entire SOTABM model is available for download from http://bionetgen.org/SCAI-wiki/index.php/Main_Page so that interested readers can view the interactions themselves.

#### Host Tissue (Self-Cells)

These cells represent the general tissue of the host. They do not move and occupy each intact grid space of the SOTABM at baseline. They contain a life variable, which determines their health state. Damage to the self-cells is reflected by a decrement of the life variable. When damaged beyond a certain threshold (arbitrarily set at <70% health, or <life = 70), the self-cells will produce damage-associated molecular pattern molecules (DAMPS) that will activate various inflammatory cells. Self-cells can be damaged by bacteria, or by the production of reactive oxygen species (r-oxy-s) from immune cells, or directly upon initialization in the sterile tissue injury mode. They are healed primarily by anti-inflammatory macrophage species (host-anti-inflam-macros), though also to a less or degree by proinflammatory macrophage species (host-pro-inflam-macros).

#### Simulated Bacteria (Bacteria-Present)

Ref. (20, 57) was used to develop the rules for bacterial infection in the SOTABM. Bacterial infection is simulated abstractly by using placeholder agents representing the presence of infection (bacteria-present), which themselves have a state variable representing the amount of bacteria present on a single patch (bacteria-count). As noted earlier, bacteria are introduced into the simulation at initialization at varying sizes of initial insult. The bacteria reduce the life of the self-cell present on their own patch, and when the life of that self-cell is reduced to 0, the bacterial colonies/clusters spread to an adjacent patch, where the subsequent value of bacteria-count on the target patch represents the magnitude of bacteria that have spread. The bacteria produce pathogen-associated molecular pattern molecules (PAMPS), which act analogously to DAMPS in terms of attracting and activating immune cells. Bacteria are killed by r-oxy-s, as well as by activated proinflammatory macrophages (host-pro-inflam-macros).

#### Transplanted Tissue (Transplant Cells)

Solid organ transplant is represented by the application of a solid section of 109 transplant cells in the right upper quadrant of the SOTABM (size arbitrarily set to 109 cells). Transplant cells perform all the functions of the self-cells in terms of keeping track of their health via the life variable and producing DAMPS if damaged. In addition, they also have a state variable for non-self antigen (transplant-Ag), which can be passed on to any host antigen-presenting cells [host macrophages (host-macros) and host-DCs] that come into contact with them. Also, in addition to being able to be damaged by bacteria or r-oxy-s, they can also be directly damaged by activated cytotoxic CD8^+^ T cells (cyto-CD8-ts).

#### Polymorphonuclear Neutrophil Cells

These are the most common type of inflammatory cells; the rules for polymorphonuclear neutrophil cells (PMNs) are drawn from Ref. (20, 52, 53, 57). They move randomly unless in the presence of their chemotactic triggers (DAMPS and PAMPS). When triggered, they follow the gradients of these molecules to the areas of injury or infection, where they undergo respiratory burst. This results in the production of r-oxy-s, which kills bacteria and damages normal tissue.

#### Host Macrophages (Host-Macros)

Their interactions are depicted under boxes “Macrophages,” “Pro-Inflam Macros,” and “Anti-Inflam Macros” in Figure 2. The rules for host-macros and their subtypes are derived from Ref. (20, 47, 52, 53, 57, 61, 62). Similar to PMNs, these immune cells move randomly unless in the presence of threshold levels of their chemotactic triggers: a combination of DAMPS/PAMPS and tumor necrosis factor (TNF). They become activated into either a proinflammatory phenotype or an anti-inflammatory phenotype depending on their milieu. DAMPS, PAMPS, and TNF all favor the proinflammatory state, while interleukin-10 (IL-10) favors the anti-inflammatory state. Proinflammatory activated macrophages (host-pro-inflam-macros) will produce both TNF and IL-10 based on their level of stimulation by PAMPS and DAMPS. The effect of IL-10 is to decrease the responsiveness of host-DCs and host-macros to recognize antigen. They will also abstractly perform phagocytosis (by reducing the bacteria-count of bacteria-present on patches colocated with the host-macro), and weakly heal normal tissue. Anti-inflammatory activated macrophages (host-anti-inflam-macros) will produce IL-10 based on their level of stimulation by PAMPS, DAMPS, and TNF; they do not produce TNF. They are the primary healing cells in the SOTABM, representing this function abstractly by increasing the life of any self-cells present until they return to normal. Also, unactivated host-macros are able to recognize non-self antigens (transplant-Ag) when they come into contact with transplant cells. Once they carry transplant-Ag, they are able to convert any naïve CD8 T cells (naïve-CD8-ts) in the lymph node area of the SOTABM to cyto-CD8-ts, which can then migrate to the area of the transplant and damage it.

#### Host Dendritic Cells (host-DCs, pro-host-DCs, and tol-host-DCs)

These cells function similarly to unactivated host-macros, and rules for their behavior were derived from Ref. (47, 63, 64). If they come into contact with bacteria or transplant cells, they will pick up either bacteria-Ag or transplant-Ag. These activated dendritic cells have two distinct paths: either their default path as proinflammatory dendritic cells (pro-host-DCs) that are able to activate naïve-CD8-ts to their cytotoxic form (cyto-CD8-ts) through direct contact, or as tolerogenic dendritic cells (tol-host-DCs) that directly inhibit the generation of cyto-CD8-ts, as well as activating T-regs and producing IL-10. This last function, the production of IL-10, is a negative feedback control mechanism that reduces the ability of host-DCs and host-macros to pick up antigen in the first place. The default trajectory of an antigen-activated host-DC is toward the pro-host-DC phenotype, but interaction with graft-MSCs will switch them to the tol-host-DC phenotype.

#### CD8^+^ T Cell Species (naïve-CD8-ts and cyto-CD8-ts)

Rules for T cells in this and the following sections were derived from Ref. (47, 49–53, 62, 63). These cells are initialized as naïve-CD8-ts in the left upper quadrant of the SOTABM, simulating their baseline existence in lymph tissue. In their naïve form, they do not move, but if they are exposed to/colocated with host-macros or host-DCs that are positive for transplant-Ag, then they become activated to cyto-CD8-ts, which can then move to the area of transplant tissue. If they come into contact with transplant cells they will reduce their life, leading to the production of DAMPS and eventually killing the transplant cell.

#### Regulatory T Cells

This agent class is used to abstractly aggregate a large set of different subtypes of T cells (many of which are CD4^+^ but also includes CD8^+^ regulatory cells, double negative CD T cells, among others) (47, 49–53, 55, 63). While a plethora of these cell types exist, in general, they share many common features:

1. Their production and function are enhanced by IL-10.
2. Many produce IL-10.
3. They inhibit the generation of, function of, and promote the apoptosis of both effector T cells and non-tolerogenic dendritic cells.
4. They promote the generation and function of tolerogenic dendritic cells.

Therefore, the current version of the SOTABM aggregates these functions into a single abstract t-regs class. T-regs freely move, reflecting their initial peripheral location. They become activated through interactions with antigen-presenting cells (either host-macros or host-DCs with positive transplant-Ag); once activated, they produce IL-10. They are also able to induce apoptosis of antigen-presenting cells already activated with transplant-Ag.

#### Mesenchymal Stromal/Stem Cells

These are immature, multipotent cells initially derived from the bone marrow but present in virtually all organ tissue (including transplanted organs); rules for implementation of mesenchymal stromal/stem cells (MSCs) are drawn from Ref. (47, 54, 55, 58, 65–67). These cells are activated by inflammation, though not immediately or acutely, as would be seen in tissue trauma or bacterial infection. Rather, their function is more pronounced in the face of longer standing inflammation, as would be seen in chronic infections or persistent inflammation. MSCs have potent anti-inflammatory properties triggered by exposure to DAMPS, resulting in the downregulation of effector T cells. Their specific role in transplant immunology is not completely clear. The SOTABM focuses on the role of MSCs only in the graft tissue (graft-MSCs) because: (1) MSCs do not appear to be present in meaningful numbers in the circulation, (2) the apparent time course of MSC activity lies outside the time period where host-derived MSCs might affect acute rejection, and (3) MSCs are present in organs commonly transplanted (i.e., liver, kidney, and heart). Graft-MSCs become activated by DAMPS, deactivate cyto-CD8-ts, and promote the generation of tol-host-DCs.

### Simulated Antirejection Immunosuppression

It is generally accepted that in the absence of immunosuppression, all non-identical genotype organ transplants will result in acute rejection (47). A primary goal of immunosuppressive therapy is to tip the balance from T-cell-mediated immunity and cytotoxicity toward a tolerogenic phenotype dominated by T-regs (59). The end effector targets of immunosuppression can be seen in the blue emphasis region in Figure 2. While there are many different specific targets for immunosuppressive drugs, this current paper is focused on evaluating the effects of reducing effector T-cell populations/function while attempting to spare the role of T-regs. The SOTABM simulates the following general classes of immunosuppression.

#### T-Cell Eradicative Therapies

These therapies, which are primarily polyclonal or monoclonal antibodies directed against T cells, are used as induction modalities (60). They are traditionally thought to function by depleting the host’s T-cell populations, reducing the initial adaptive immune cellular response to the graft, and favoring the generation of tolerogenic, T-reg populations, though they are more recently recognized as having additional effects related to interference with leukocyte–endothelial adhesion as well as reducing dendritic cell function (60). For simplicity’s sake, this initial version of the SOTABM focuses on simulating only the effect of T-cell depletion and represents this effect by allowing for 90% percentage depletion of T cells from day 2 to day 14 following transplant (68).

#### Calcineurin Inhibition

Calcineurin is a phosphatase that dephosphorylates the transcription factor necessary for T-cell activation (nuclear factor for the activation of T cells or NFAT) and allowing its localization in the nucleus. Inhibition of calcineurin prevents this localization and limits the activation of T cells. The SOTABM uses data regarding two of the most commonly used calcineurin inhibitors, cyclosporine A and tacrolimus, as reference points for the simulation of calcineurin inhibition (69–72). While both of these compounds block T-cell activation arising from interleukin-2 (IL-2) responsiveness, in the interest of simplicity the dynamics of IL-2 are not explicitly modeled. As such, the SOTABM qualitatively simulates the effect of calcineurin inhibition by reducing the probability that both naïve-CD8-ts are converted to cyto-CD8-ts, as well as the activation of T-regs by tol-host-DCs.

#### Cell-Based Supplementation Therapies

There is increasing interest in providing organ transplant patients with supplementary populations of those cells believed to favor the tolerogenic phenotype. T-regs and regulatory macrophages have been employed in this fashion, with initially promising results (47). However, the scalability of these modalities is hampered by practical barriers in the collection/generation of sufficient populations of appropriately configured cells. Therefore, cell transfer research has naturally turned toward those cell types that may be more readily available. Specifically, mesenchymal stromal cells have been employed, with varying results (47, 54, 58). The SOTABM simulates the effect of MSC transfer therapy by the addition of 100 MSCs to the simulation following application of the transplant.

## Simulation Experiments

As noted earlier, current version of the SOTABM is intended as an initial example of an ABM that can serve as a scalable framework for dynamic knowledge representation of transplant immunology. The goal of such initial simulation experiments is to provide face validity, i.e., sufficiently plausible model behavior reflected in the qualitative mapping between the simulation output and the real-world behavior (6, 42, 43). This modeling goal places the current version of the SOTABM in the earliest phases of the POM process (14, 15). It should be noted that there is not a presumption of “uniqueness” of this particular configuration of the SOTABM. Rather, achieving face validity with the current iteration of the SOTABM just demonstrates that there exists a configuration of model parameters such that these behaviors can be reproduced (3, 6, 21). As applied to the SOTABM, this approach leads to the execution of simulation experiments aimed at replicating the conditions listed below.

Simulation experiments utilize the stochastic nature of ABMs to generate simulated populations for each experiment performed with the SOTABM. The following experiments were performed with *N* = 100 incidences per condition:

- Baseline immune response to injury and infection: these simulations were performed to establish plausible behavior of the SOTABM in terms of its ability to recover initial perturbations involving just tissue damage (sterile injury) or infection. “Death” of the system was arbitrarily defined as when the simulation run reached a level <20% total system health (reflected by the summed life variables of all the self-cells). Simulations were performed reflecting 28 days of simulated time and consisted of a *parameter sweep* of the level of the initial insult. A parameter sweep consists of a series of simulation runs (*N* = 100) across a range of the selected parameter. In this case, the parameter is the initial amount of injury (initial injury number) or infection (initial infection number) applied to the SOTABM, and the parameter sweeps performed can be considered analogous to the dose–response range or generated mortality in the design of a particular wet-lab experimental model. Plausible behavior would be reflected by bounds on the ability of the system to survive based on the magnitude of the initial insult, below which where survival = 100% and above which survival = 0%. This is similar to the previously utilized method for evaluating the response of an ABM of systemic inflammation to injury and infection (19, 20).
- Baseline immune response to transplant: as opposed to the simulation experiments used to examine SOTABM response to injury and infection, which consisted of parameter sweeps of the magnitude of initial perturbation, the amount of transplanted tissue applied is fixed (109 contiguous transplant cells in a roughly square configuration). Since all transplanted organs undergo some degree of damage, a “successful” transplant was viewed as the simulation having >20% of the transplanted tissue remaining (as reflected by the sum of the life variable of all the) after 1 year of simulated time (arbitrary percentage). Plausible behavior would consist of loss of all transplant tissue by the end of 1 year simulated time in the absence of immunosuppression (73).
- Simulation of immunosuppressive therapies: as noted in Section “Methods,” the SOTABM has the capability to simulate several antirejection therapies.
  ○ T-cell eradicative therapy is simulated by the deactivation of 90% of all T-cell agents on day 2 post-transplant extending to day 14 post-transplant, at which time T-cell populations were allowed to recover. This rule was adapted from Ref. (60, 68), with an exclusive focus on the effect of anti-T-cell antibody therapy with respect to decreasing T-cell populations at doses approximately corresponding to use in human organ transplant.
  ○ The effect of calcineurin inhibition is simulated by the reduction of the probability that naïve-CD8-ts are converted to cyto-CD8-ts to 10% per encounter, while the effective preserving activation of T-regs by tol-host-DCs consists of having activation occur at a probability of 80% per encounter; this effect was persistent during the 1 year of simulated time, reflecting the continued use of the therapeutic agent. These effects and values were extrapolated from information extracted from Ref. (69–72).
  ○ Simulation of cell transfer therapy using MSCs was simulated by the addition of 100 MSCs to the simulation following application of the transplant (58). This number of MSCs, relative to the number of PMNs (=200 at initialization) in the SOTABM, is within the range (lower end) of *in vivo* studies investigating MSC transfer therapy (58). PMNs were chosen as the reference cell population number due to the greater availability of their circulating numbers.

Simulation experiments of immunosuppressive strategies consisted of each intervention alone (parsed interventions), T-cell eradicative therapy plus calcineurin inhibition (approximation of current clinical practice), and T-cell eradicative therapy plus calcineurin inhibition plus MSC transplant (hypothetical). Parsed immunomodulation simulations are considered component testing for the SOTABM’s simulation of immunosuppression. However, since clinical data do not exist for such interventions in isolation, the SOTABM’s output can only be viewed in the most qualitative fashion aimed at producing plausible results. The clinical reference outcome focuses on 1-year graft survival in the T-cell eradication + calcineurin inhibition group, which most closely approximates current standard clinical practice. Given the fact that the SOTABM utilizes a generic transplanted tissue, reference values were drawn from a range of SOTs: specifically kidney, liver, and heart. The reference range for renal transplant (cadaveric, due to the allogenic nature of the generic transplanted tissue in the SOTABM) was a 1-year graft survival range of 89–91% (74). The reference range for hepatic transplant was a 1-year graft survival of 71–80% (the range representing the difference between deceased cardiac donors and deceased brain donors, a distinction not within the SOTABM’s current representational capacity) (75). The reference range for cardiac transplant was a 1-year graft survival of 83–89% (range reflecting stratification of high to low risk transplants in a study on the effect of case volume on outcome) (76). While there are several ongoing clinical trials for MSC transfer, explicit data for 1-year graft survival currently do not exist for this intervention (47, 54, 58, 59); therefore, the “hypothetical” condition of MSC transfer + T-cell eradication + calcineurin inhibition is considered a prediction pending the reporting from those trials.

## Results

### Baseline Immune Response to Injury and Infection

**Figure 3:**
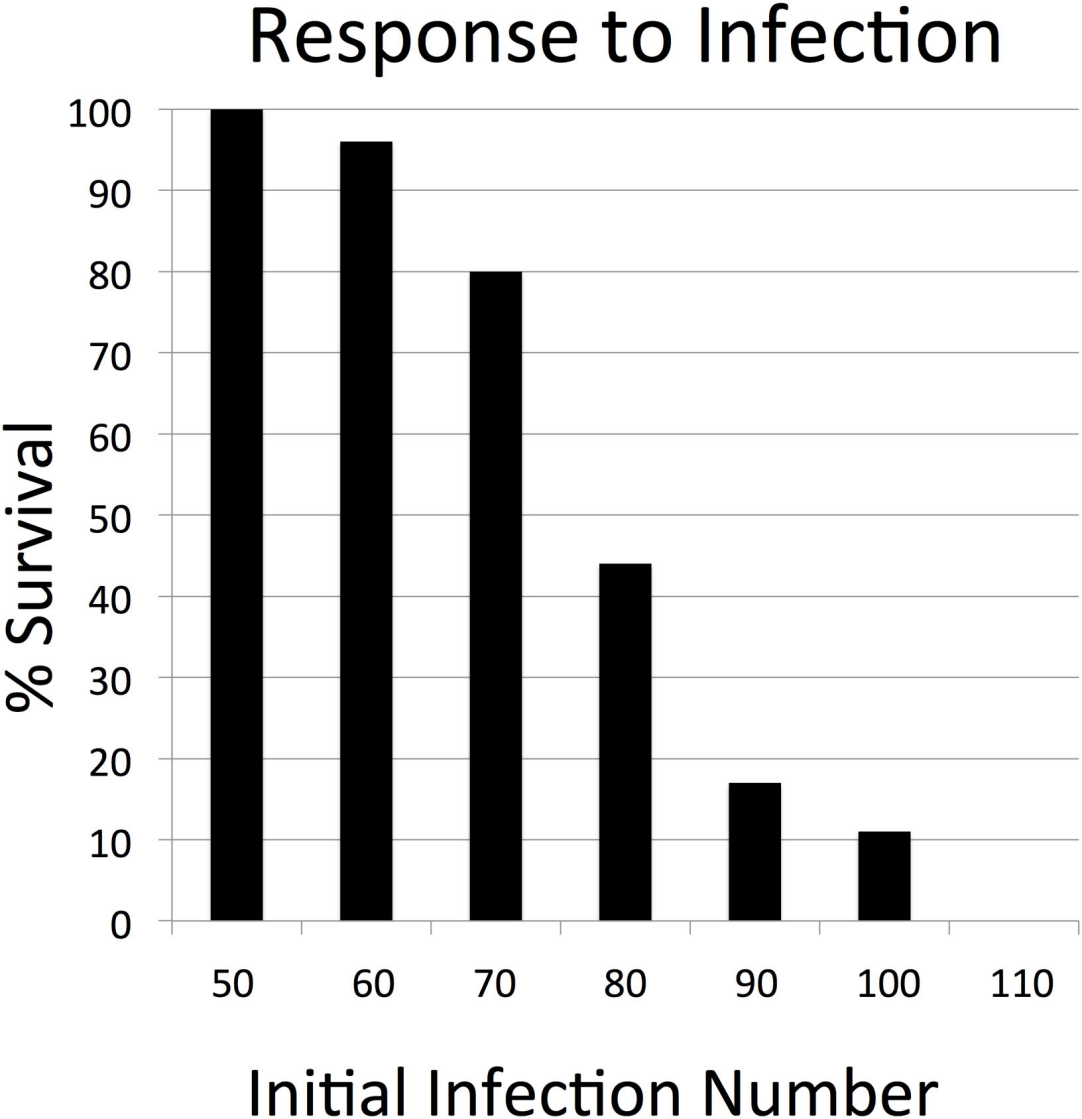
**Parameter sweep of the SOTABM for infection. This figure depicts the transition zone from 100% survival (below initial infection number = 50) toward 0% survival (above initial infection number = 110)**. These results demonstrate that the response of the SOTABM to infection is appropriately and plausibly bounded, meaning that there was an initial infection number below which the system always healed and an initial infection number above which the system always died. There were 100 replicates (*N* = 100) for each condition simulated, simulated time = 28 days.

**Figure 4:**
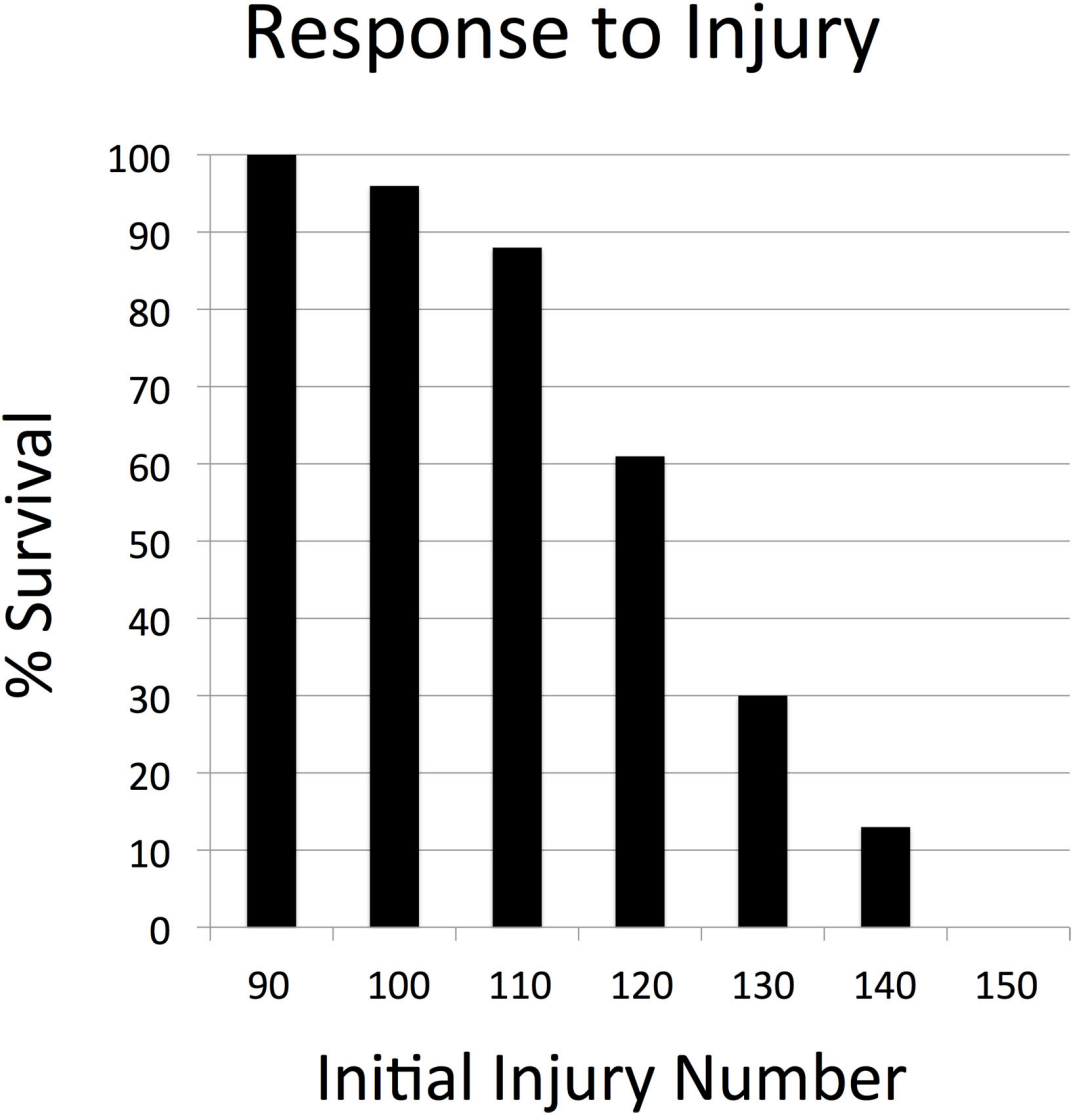
**Parameter sweep of the SOTABM for tissue injury. This figure depicts the transition zone from 100% survival (below initial injury number = 90) toward 0% survival (above initial injury number = 150)**. These results demonstrate that the response of the SOTABM to tissue trauma is appropriately and plausibly bounded. *N* = 100 for each condition simulated, simulated time = 28 days. The behaviors displayed in Figures 3 and 4 with respect to non-transplant conditions where inflammation and immune responses are recognized to occur serve as verification points for the SOTABM.

In the absence of any perturbation, the cell levels and tissue integrity of the SOTABM were dynamically stable, as would be expected. The results of the parameter sweeps of initial perturbation demonstrated plausible behavior for both sterile tissue injury and infection. Simulated infection demonstrates an initial inflection point with respect to the transition from complete survival at initial infection = 50 (survival = 100%), with progressively worsening likelihood of survival at increments of 10 of initial infection until reaching a second point, beyond which there is always system death (initial infection = 110 with survival = 0%); see Figure 3. Similarly, with respect to sterile injury, the earlier transition point from complete recovery (100% survival) was at initial injury = 90, with an upper transition into complete lethality (0% survival) at initial injury = 150 (see Figure 4).

### Baseline Immune Response to Transplant

As expected, there were no simulation runs with transplant tissue survival at the end of 1 year simulated time; the average time to critical transplant tissue loss was 14.6 days of simulated time, with the longest transplant survival ~21 days. This is slightly greater that the recognized timeframe of 10–13 days for cell-mediated tissue graft rejection, but not vastly so (73).

### Simulation of Immunosuppressive Therapies

**Figure 5:**
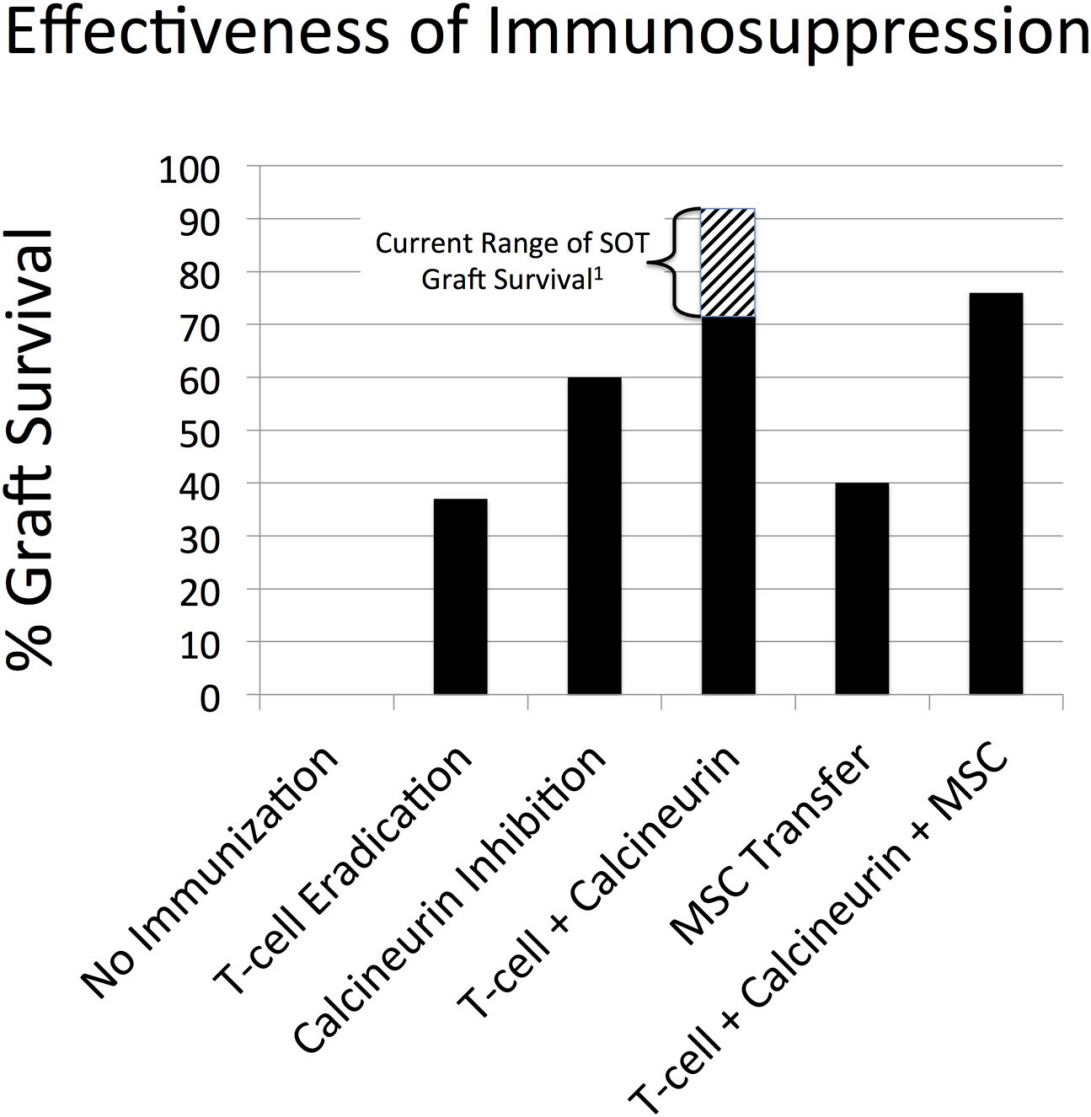
**Effectiveness of different immunosuppression simulations in the SOTABM**. This figure depicts the results of first no immunosuppression, then the following simulated immunosuppressive therapies alone and in combination: T-cell eradicative therapy alone = 37% graft survival (note no re-dosing for episodes of acute rejection); calcineurin inhibition = 60% graft survival; combination therapy of T-cell eradication with calcineurin inhibition = 72% graft survival; MSC transfer alone = 40% graft survival (no re-dosing but immortal MSCs); and T-cell eradication plus calcineurin inhibition plus MSC transfer = 76% graft survival. *N* = 100 for all conditions, total simulated time = 1 year. Note: ^1^Aggreated solid organ transplant (SOT) 1-year graft survival range of 71–91% incorporating outcomes from renal, hepatic, and cardiac transplants (74–76).

These results are depicted in Figure 5. There were 100 replicates (*N* = 100) for all conditions, with the total simulated time represented by a simulation run = 1 year. The results of the simulated immunosuppressive therapies are as follows:

- Parsed modalities: T-cell eradicative therapy alone = 37% graft survival (note no re-dosing for episodes of acute rejection); calcineurin inhibition alone = 60% graft survival; and MSC transfer alone = 40% graft survival (no re-dosing but immortal MSCs). As noted earlier, since corresponding clinical data does not exist for each of these therapeutic interventions in isolation, the results of the SOTABM can only be evaluated in a highly qualitative fashion. Given this limitation, each modality plausibly has some beneficial effect on 1-year graft survival, but with a plausibly lower efficacy than combination therapy approximating current practice.
- Approximated current therapy: combination therapy of T-cell eradication with calcineurin inhibition = 72% graft survival; compare to a range of 1-year graft survival of 71–91% incorporating outcomes from renal, hepatic, and cardiac transplants (74–76). Simulation 1-year graft survival was lower than reported clinical rate. However, this can be explained by the fact that the current set of simulated immunosuppressive regimens did not allow for re-dosing of immunosuppression for episodes of acute rejection, as would be the case in the clinical situation.
- Hypothetical MSC transfer: T-cell eradication plus calcineurin inhibition plus MSC transfer = 76% graft survival. As noted earlier, there currently does not exist an appropriate data set for comparison. The slight increase in 1-year graft survival is plausible but must remain only a prediction from the SOTABM pending the reporting of the outcomes of the ongoing clinical trials.

## Discussion

The most fundamental goal of biomedical research is to develop the ability to effectively and beneficially control the trajectory between health and disease; in short, the practice of medicine is a control problem. The ability to exercise control requires a putative mechanism by which the control can be exercised, and this in turn requires an understanding of the overall and aggregate structure in which the individual mechanisms reside. Furthermore, effective control does not necessarily require comprehensive knowledge of the system being controlled; what is required is a sufficiently detailed representation of the target system such that a control strategy can be developed and potentially tested. The use of selective abstraction is fundamental to the scientific process: it is universally utilized as a means of gaining insight, increasing the general applicability of acquired knowledge, and from a practical standpoint, identifying what constitutes actionable knowledge. Unfortunately, there is a general paucity of abstract thinking in biology; a situation with historical and cultural antecedents (41). Therefore, the current challenge that faces the biological (and biomedical) research communities is gaining the ability to facilitate abstract representations of mechanistic knowledge in order to best leverage the vast sets of data that are currently being generated. It has been proposed that dynamic computational models, and ABMs in particular, can aid in affecting this translational goal (22, 39).

The SOTABM is an initial step at developing an agent-based modeling framework to do this for transplant immunology, representing a highly abstracted model of the components and processes of the immune response, upon which various perturbations, including transplant, can be applied. The design philosophy of the SOTABM emphasizes its ability to represent a greater range of conditions (i.e., response to infection, tissue injury, and transplant challenge) rather than striving for “precision” with respect to replicating a specific disease process. This, in fact, is how biological systems function: they have an underlying set of functions that have been acted upon by evolution in a selection process that favors the ability to deal with heterogeneous, disparate, and potentially novel conditions. Without this ability to have conserved core functionality, evolution of biological systems could not occur. Therefore, this initial presentation of the SOTABM emphasizes the ability of the system to recover from a range of perturbations, rather than necessarily trying to create a highly detailed representation aimed specifically at simulating solid organ transplantation. As such, the SOTABM utilizes the minimally sufficient control structure that maps to the biological system while being able to produce the desired behavioral features. Once this iteration of the model is deemed sufficient, the next step is to identify features of the reference system that are not adequately represented; at this point, additional detail is added to the model. This is the iterative refinement process defined by Hunt et al. (6, 21) and represents a model design and development strategy that is consistent with the Popperian paradigm that science progresses via sequential falsification.

The current version of the SOTABM generally, plausibly, and qualitatively reproduces the inflammatory/immune system response to different types of perturbations while incorporating a set of minimally detailed components and primary features necessary in characterizing early adaptive immunity. The simulation experiments concerning the response of the SOTABM to infection and injury represent plausibility checks, particularly since the cellular–molecular control structure represented in the SOTABM arose through evolution to meet these types of perturbations. Put a different way, a model that solely focuses on the response to transplant would have limited biological plausibility, since the evolutionary forces that led to the development of the control structure would not be accounted for. It is only in the context of a model that does produce plausible responses to evolutionarily relevant conditions that the behavior of the model in response to an “artificial” situation (i.e., SOT) can be reasonably assessed. The fact that even given its high degree of abstraction and biological incompleteness the SOTABM is able to generate qualitatively plausible responses to a range of perturbation points to a fundamental soundness of the knowledge representation incorporated into the model. However, there are clear limitations to the current version of the SOTABM, several of which are listed below:

- As a model created in Netlogo, the SOTABM inherits the limitations associated with that modeling environment, as would be the case with any computational model of virtually any form. The benefits of Netlogo are that it has a very low initial threshold for use, possessing an excellent tutorial and a robust library of example models; it allows a novice modeler to fairly rapidly engage in the model-creation process. However, this ease-of-use carries with it a set of hidden dangers, most prominently related to the fact one can readily fall into the trap that with increasing facility in the use of the tool one starts to think of their reference system in primarily terms of that tool, rather than as a subject in of itself. This is not an issue unique to NetLogo, rather it is a pervasive issue that not only affects computational modeling but also affects experimental research, where the tools for investigation begin taking precedence and coloring the interpretation of the reference system itself. The aphorism describing this phenomenon is: “To one with a hammer everything looks like a nail.” The solution this challenge is the use of cross platform validation, where the underlying conceptual model is implemented in a set of different, ideally unrelated modeling methods. Full discussion of this issue is beyond the scope of this paper, except to say that this is an issue that the modeling community struggles with, and where recognition of the challenge is currently the best, most prudent strategy.
- Moving on to specific limitations of the SOTABM within the context of its development environment, the lack of adjustment of therapeutic regimen to treat episodic acute rejection. In the clinical setting, there is considerable surveillance looking for signs of early rejection, and these episodes are addressed with temporary augmentation of the immunosuppressive regimen. The current cycle of simulation experiments do not reflect this practice.
- Lack of sufficient model detail with respect to the mechanisms of immunosuppression, While above we have made the argument concerning the benefits of abstraction, there is a definite point at which the failures of a particular abstraction level become evident. This may be most pronounced when dealing with putative mechanisms of control. In the case of simulated immunosuppression in the SOTABM, the abstractions made with respect to the life cycle of immune cells, and the various stages of activation possibly resulted in a too-coarse graining of responses the system; in short the abstractions made enforced a more binary, and less nuanced, set of possible trajectories for the different cellular populations and their activation status.
- Solid organ transplant does not occur without tissue trauma from the initial surgery. In fact, there can be a huge variation in the amount of surgical trauma/resuscitation associated with a transplant, which in turn is due to a large amount of variance in the presurgical morbid state of the patient. These factors clearly influence the success of the overall transplant, but due to the inherent interactive complexity of this clinically relevant condition, it is essentially impossible to parse out how each of those factors might actually come into play for a particular individual. A suggestion for that process of parsing is the goal of this initial paper: by decomposing the different possible functional components of the overall transplant patient, the sets of conditions presented here should be thought of as semi-idealized, reductionist interpretations of the admittedly complex system dynamics. The process is analogous to the rationale for using simpler, reduced biological proxy experimental platforms to do research (cell cultures, tightly controlled animals, etc.), but with significant and critical differences. The first of these differences is the fact that computational models are transparent with respect to the mechanisms being evaluated: there are no “hidden variables” (i.e., biological components or functions). This means that ABMs will only do what is put into them, and therefore their failure to be made to generate a desired behavior is direct evidence of their insufficiency (thereby achieving the goal of falsification). The second difference is that their transparent, modular structure allows ABMs to be aggregated (perhaps “reverse parsed”) in a fashion that is not currently feasible in experimental biology. While there are several steps in this direction (i.e., linked “organs-on-a-chip”), the current translational step from *in vitro* to *in vivo* experimental platforms is opaque to a whole host of processes and interactions that cannot be identified or characterized.

Recognizing the costs of the abstractions and omissions made in the current version of the SOTABM provides a guide for the necessary refinements to be made in its next iterations. Importantly, the ability to modularly extend the SOTABM to more closely match the richness of the cellular subtypes and response capabilities in the early adaptive immune response is one of its key intended features. It is hoped that this initial implementation of the SOTABM will demonstrate its promise as a framework that can serve to integrate the continually evolving knowledge concerning transplant immunity and help fulfill the promise of dynamic knowledge representation as a means of addressing the Translational Dilemma.

## Conflict of Interest Statement

The author declares that the research was conducted in the absence of any commercial or financial relationships that could be construed as a potential conflict of interest.
